# Supplementary material for: Transcriptional Repression of CCL2 by KCa3.1 K+ Channel Activation and LRRC8A Anion Channel Inhibition in THP-1-Differentiated M2 Macrophages
Source: Int J Mol Sci. 2025 Aug 6;26(15):7624. doi: 10.3390/ijms26157624 (PMC12347553; doi:10.3390/ijms26157624)
Supplement: Supplementary file 1 [file ijms-26-07624-s001.zip › ijms-3693196-supplementary.pdf]

**Supplementary Materials**

# Transcriptional Repression of CCL2 by $K_{Ca}3.1$ $K^+$ Channel Activation and LRRC8A Anion Channel Inhibition in THP-1-Differentiated $M_2$ Macrophages

**Miki Matsui †, Junko Kajikuri †, Hiroaki Kito, Yohei Yamaguchi and Susumu Ohya ***

Department of Pharmacology, Graduate School of Medical Sciences, Nagoya City University, Nagoya 467-8601, Japan; c241739@ed.nagoya-cu.ac.jp (M.M.); kajikuri@med.nagoya-cu.ac.jp (J.K.); kito@med.nagoya-cu.ac.jp (H.K.); y_yamagu@med.nagoya-cu.ac.jp (Y.Y.)

* Correspondence: sohya@med.nagoya-cu.ac.jp; Tel.: +81-52-853-8149

† These authors contributed equally to this work.

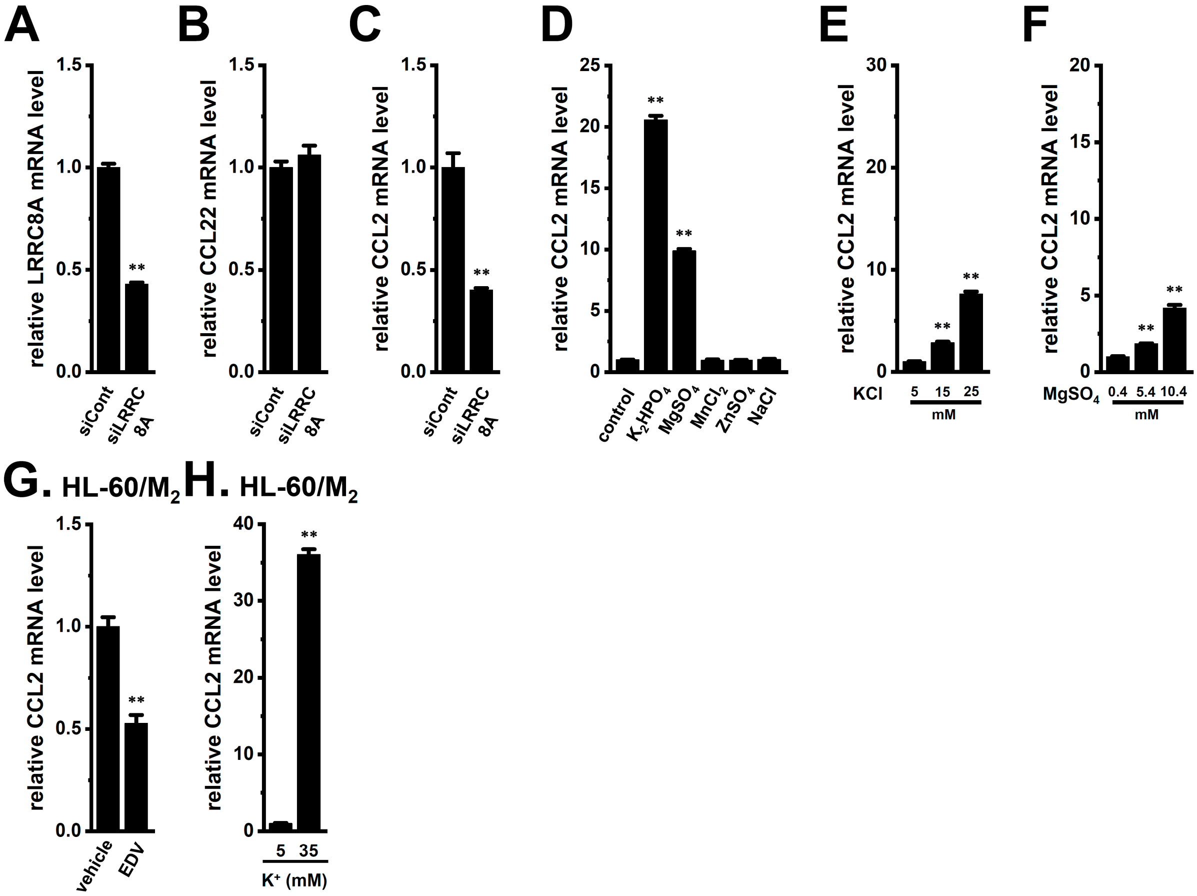


**Figure S1.** Effects of siRNA-mediated LRRC8A inhibition and treatment with various metal ions on CCL2 expression in $M_2$-MACs and effects of treatment with EDV and exposure to high $[K^+]_e$ on it in HL-60-derived $M_2$-like macrophages. **A-C**: Real-time PCR examination of LRRC8A (**A**), CCL22 (**B**), and CCL2 (**C**) expression in $M_2$-MACs transfected with siCont or siLRRC8A for 48-hr (*n* = 4). **D**: CCL2 expression in $M_2$-MACs treated with various salts ($K_2HPO_4$, $MgSO_4$, $MnCl_2$, $ZnSO_4$, and NaCl) for 12-hr (*n* = 4). **E**, **F**: CCL2 expression under increasing concentrations of $[K^+]_e$ (**E**) or $[Mg^{2+}]_e$ (**F**) (*n* = 4). **G, H**: CCL2 expression in HL-60-derived $M_2$-like macrophages under LRRC8A inhibition or high $[K^+]_e$ (*n* = 4). **: $P < 0.01$ vs. siCont, vehicle control, or normal $[K^+]_e$ / $[Mg^{2+}]_e$.

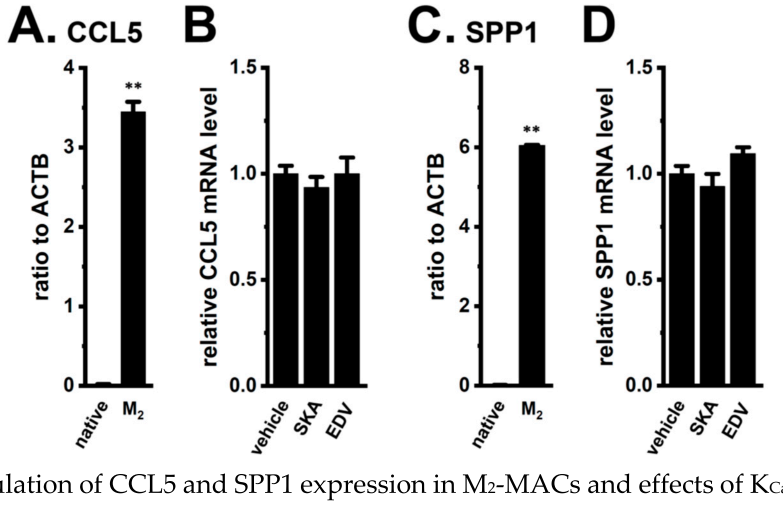


**Figure S2.** Upregulation of CCL5 and SPP1 expression in $M_2$-MACs and effects of $K_{Ca}3.1$ activation and LRRC8A inhibition on them. **A, C**: CCL5 (**A**) and SPP1 (**C**) expression in native THP-1 cells (native) and $M_2$-MACs ($M_2$) (*n* = 4). **B, D**: CCL5 (**B**) and SPP1 (**D**) expression in $M_2$-MACs treated with vehicle, SKA, or EDV for 12-hr (*n* = 4). Expression levels are normalized to ACTB. **: $P < 0.01$ vs. native THP-1.

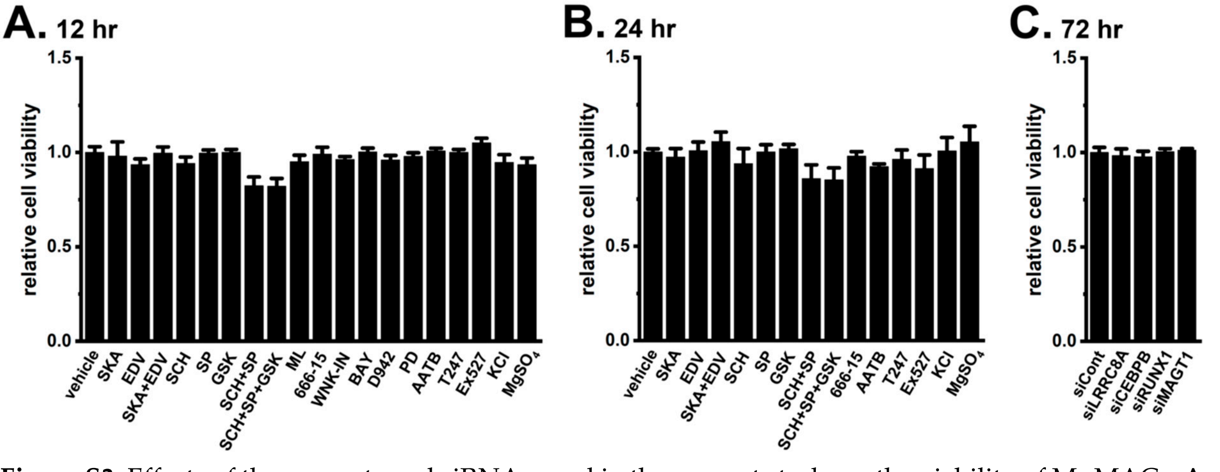


**Figure S3.** Effects of the reagents and siRNAs used in the present study on the viability of $M_2$-MACs. **A, B**: Effects of the treatment with 10 μM SKA121 (SKA), 10 μM endovion (EDV), SKA+EDV, 1 μM SCH772984 (SCH), 1 μM SP600125 (SP), 10 μM GSK2795039 (GSK), SCH+SP, SCH+SP+GSK, 5 μM ML385 (ML), 1 μM 666-15, 1 μM WNK-IN-11 (WNK-IN), 1 μM BAY3827 (BAY), 1 μM D942, 1 μM PD169316 (PD), 10 μM AATB, 10 μM T247, 1 μM Ex527, 30 mM KCl, and/or 20 mM $MgSO_4$ for 12- (**A**) or 24- (**B**) hr on the viability of $M_2$-MACs using the WST-1 assay ($n$ = 5). **C**: Effects of the siRNA-mediated inhibition of LRRC8A (siLRRC8A), CEBPB (siCEBPB), RUNX1 (siRUNX1), and MAGT1 (siMAGT1) on the viability of $M_2$-MACs ($n$ = 5). Cell viability in the vehicle-treated and control siRNA (siCont)-transfected groups was expressed as 1.0. Two hours after the addition of WST-1 reagent to each well, absorbance was measured using the microplate reader SpectraMax 384 at a test wavelength of 450 nm and a reference wavelength of 650 nm [14].

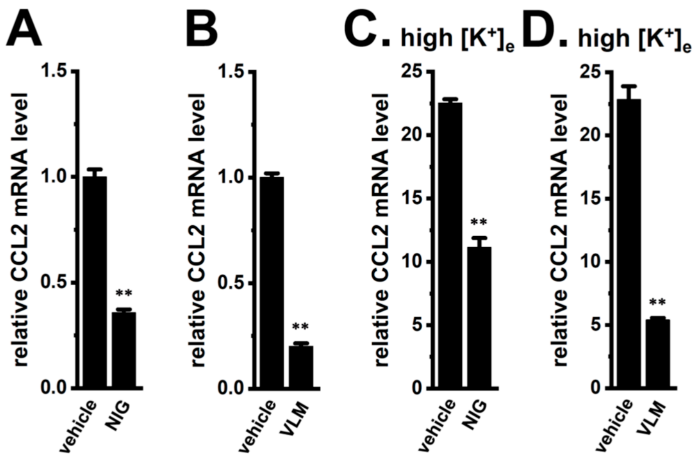


**Figure S4.** Effects of two $K^+$ ionophores on CCL2 expression under normal and high $[K^+]_e$ conditions in $M_2$-MACs. **A-D**: Real-time PCR examination of CCL2 expression in $M_2$-MACs treated nigericin (NIG, 1 μM) (**A, C**) and valinomycin (VLM, 1 μM) (**B, D**) for 12-hr under normal (**A, B**) and high (**C, D**) $[K^+]_e$ conditions ($n$ = 4). In **C** and **D**, expression levels are shown relative to normal $[K^+]_e$ conditions (set as 1.0). $^{**}$: $P < 0.01$ vs. vehicle control.

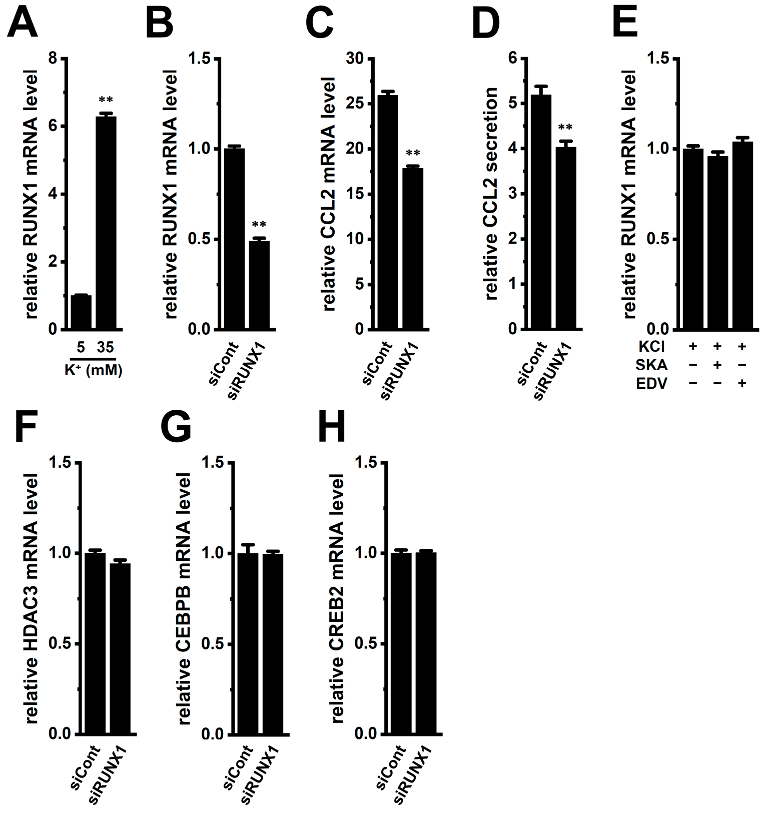


**Figure S5.** Upregulation of RUNX1 in M2-MACs exposed to high $[K^+]_e$ and effects of $K_{Ca}3.1$ activation and LRRC8A inhibition on its expression. **A**: RUNX1 mRNA expression in M2-MACs exposed to high $[K^+]_e$ for 12-hr ($n$ = 4). **B**: Knockdown efficiency of RUNX1 siRNA (siRUNX1) after 48-hr transfection in M2-MACs. mRNA levels are shown relative to control siRNA (siCont) ($n$ = 4). **C, D**: Effect of siRNA-mediated inhibition of RUNX1 (siRUNX1) on CCL2 mRNA expression (**C**) and secretion (**D**) in M2-MACs ($n$ = 4). **E**: RUNX1 mRNA expression in M2-MACs treated with 30 mM KCl (+/–/–), KCl + SKA (+/+/–), or KCl + EDV (+/–/+) for 12-hr ($n$ = 4). **F-H**: Effect of siRUNX1 on HDAC3 (**F**), CEBPB (**G**), and CREB2 (**H**) mRNA levels in M2-MACs ($n$ = 4). **: $P < 0.01$ vs. normal $[K^+]_e$ and siCont.

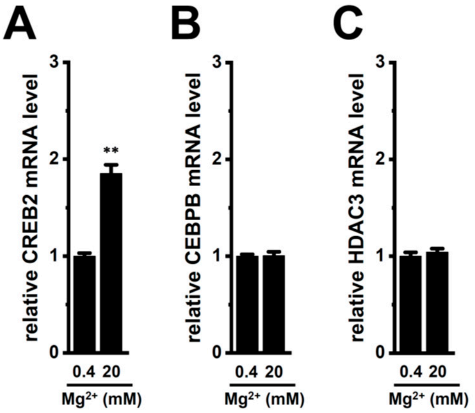


**Figure S6.** Effects of high $[Mg^{2+}]_e$ exposure on the expression of CREB2, CEBPB, and HDAC3 in M2-MACs. **A-C**: Real-time PCR examination of CREB2 (**A**), CEBPB (**B**), and HDAC3 (**C**) expression in M2-MACs exposed to 20 mM $MgSO_4$ for 12-hr ($n$ = 4). **: $P < 0.01$ vs. normal $[Mg^{2+}]_e$.

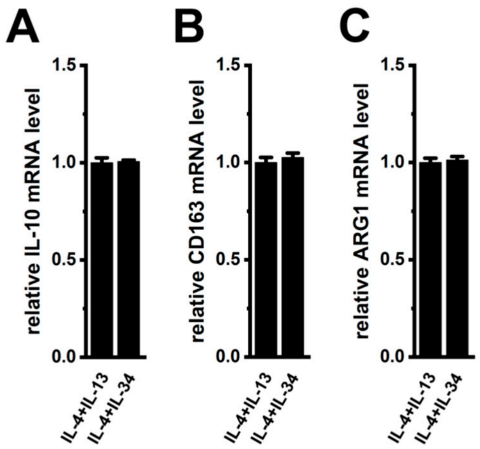


**Figure S7.** Expression of M2 markers in IL-4/IL-34-treated macrophages. **A-C**: Real-time PCR examination of IL-10 (**A**), CD163 (**B**), and ARG1 (**C**) expression in M2-MACs treated IL-4 + IL-13 and IL-4 + IL-34 (at 20 ng/mL for each for 72-hr) ($n$ = 4).

**Table S1.** List of PCR primers used in this study, related to Section 4.3.

| Target name | Genbank accession number | Primer sequence | | Amplicon length (bp) |
|---|---|---|---|---|
| | | Forward (5' to 3') | Reverse (5' to 3') | |
| CCL2 | NM_002982 | AAGACCATTGTGGCCAAGGA | GGGTTGTGGAGTGAGTGTTCAA | 120 |
| CCL22 | NM_002990 | GCGCGTGGTGAAACACTTCT | TCTGGGATCGGCACAGATCT | 100 |
| CCL5 | NM_002985 | CGTGCCCACATCAAGGAGTA | CTCCCGAACCCATTTCTTCTC | 120 |
| SPP1 | NM_001040058 | GGCATCACCTGTGCCATACC | CTTCTGAGATGGGTCAGGGTTT | 120 |
| CEBPB | NM_001285878 | GCCCTCGCAGGTCAAGAG | TGCGCACGGCGATGT | 107 |
| HDAC1 | NM_004964 | GGCTGGCAAAGGCAAGTATTA | ACTAGGCTGGAACATCTCCATTAC | 117 |
| HDAC2 | NM_001527 | GTCAAGGAGGCGGCAAAA | GGGTCATGCGGATTCTATGAG | 108 |
| HDAC3 | NM_003883 | AGAGAGTGGCCGCTACTACTGT | GCACGTGGGTTGGTAGAAGTC | 121 |
| HDAC11 | NM_024827 | AGCGTGTGTACATCATGGATGT | TCCAGGTACTCATCATCCTGTGT | 121 |
| SIRT1 | NM_012238 | ATTTTCCATGGCGCTGAGGTA | CCTCCATGGGTTCTTCTAAACTTG | 121 |
| CREB1 | NM_004379 | TGCCACATTAGCCCAGGTATC | GCCGCCTGAATGACTCCAT | 120 |
| CREB2/ATF4 | NM_001675 | GTGGCCAAGCACTTCAAACC | GAAGGCATCCTCCTTGCTGTT | 120 |
| CREB3 | NM_006368 | CCAGGCCATGGTGATTGAG | CCCCCTTGTGTCAGAGGAGTA | 121 |
| CREB3L1 | NM_052854 | CTGTTCAGCAGCTTCTTTGATGA | GTCGCCGCTCAGGGAGTAG | 120 |
| CREB3L2 | NM_194071 | GGTGATGGGCAAGGTTTCTC | GGCCCGTAGCCTTGAAAGA | 120 |
| CREB3L3 | NM_032607 | TACCTGAAGCCTCTGTGACCATAG | GGTCTTTCACGGTGAGATTGC | 120 |
| CREB3L4 | NM_130898 | GCCCAGACCAGCACTTGTGT | TCCGTGAGGCTGGTAATCCT | 120 |
| CREB5 | NM_182898 | GCCATGCAGAAAGAATCACAAG | CGATGAGGAAGTAGTGATGGTATTAT | 120 |
| ATF2 | NM_001880 | CAAACCATGCCTGTTGCTATTC | AGGACCTGGGATTCCTGGAA | 120 |
| IL-8 | BC013615 | CACTGCGCCAACACAGAAAT | TGAATTCTCAGCCCTCTTCAAAA | 120 |
| IL-10 | NM_000572 | GGCGCTGTCATCGATTTCTT | AGATGTCAAACTCACTCATGGCTTT | 120 |
| TRPM6 | NM_017662 | AGCTGTCTACCTCTTCGTGCAAT | GCGATAGCGGTTGTATTTCCA | 121 |
| TRPM7 | NM_017672 | CAGGTCATGGCCCGTTTTT | ATCATCTACCAGGTCACTCTGCTTT | 120 |
| MAGT1 | NM_001367916 | GCCTCTGCCCAAAGAAAGAA | TTTCACAAGGCGACGGAACT | 120 |
| SLC41A1 | NM_173854 | GGAATGCCCGGAGAGAACTC | TGGGACCACGAGGAGGAA | 120 |
| CNNM1 | NM_020348 | GGGACCCTTTCTATGAGGTGATG | CCCTCTGCTTTTTCCGATTG | 120 |
| CNNM2 | NM_017649 | AAGCGAGCGCCTTCTCATAC | CACCTGGAGAACCTGCTGCTA | 120 |
| CNNM3 | NM_017623 | GCTTCCTGTCCCGAGAAGTG | GCCGGTTGCTCTCGTCAA | 120 |
| CNNM4 | NM_020184 | GGTGCTGTCGGGCATATTTT | TCAATCTTGCGGGCATAGC | 120 |
| LRRC8A | NM_001127244 | ACCCCAACTCCACCATTCTG | ACCAGTGCAGTCGGTTCTCAT | 120 |
| CSF1R | NM_005211 | TGAGCAAGACCTGGACAAGGA | TCCCGGTGGATGCAATTCT | 120 |
| RUNX1 | NM_001754 | CCTCAGGTTTGTCGGTCGAA | GGCCCATCCACTGTGATTTT | 120 |
| ARG1 | NM_001244438 | ACAAAACAGGGCTACTCTCAGGAT | CCGAGCAAGTCCGAAACAAG | 131 |
| CD163 | NM_004244 | GGTCGCTCATCCCGTCAGT | TGCAAGCCGCTGTCTCTGT | 120 |
| ACTB | NM_001101 | AGGCCAACCGCGAGAAGATG | GCCAGAGGCGTACAGGGATA | 101 |

**Table S2.** List of antibodies used in this study, related to Sections 4.4 and 4.7.

| Type | Antibody name | Host species | Working dilution | Company | Product code | Observed MW (approx. kDa) |
|---|---|---|---|---|---|---|
| Primary antibody | Phospho-Nrf2 (Ser40) | rabbit monoclonal | 1:100 (ICC) | Abclonal (Tokyo, Japan) | AP1133 | |
| | Nrf2 | rabbit polyclonal | 1:100 (ICC) | Abclonal (Tokyo, Japan) | A21508 | |
| | CEBPB | rabbit polyclonal | 1:5000 (WB) | ProteinTech (Rosemont, MO, USA) | 23431-1-AP | 36 |
| | Phospho-WNK1 (Thr60) | rabbit monoclonal | 1:600 (WB) | R&D Systems (Minneapolis, MN, USA) | MAB47290 | 280 |
| | WNK1 | rabbit polyclonal | 1:2000 (WB) | ProteinTech (Rosemont, MO, USA) | 28357-1-AP | 280 |
| | Phospho-AMPK (Thr172) | mouse monoclonal | 1:600 (WB) | BioLegend (San Diego, CA, USA) | 600651 | 65 |
| | AMPK | mouse monoclonal | 1:1000 (WB) | BioLegend (San Diego, CA, USA) | 600551 | 65 |
| | HDAC3 | rabbit polyclonal | 1:750 (WB) | Santa Cruz Biotechnology (Dallas, TX, USA) | sc-11417 | 50 |
| | CREB2 (ATF4) | rabbit polyclonal | 1:1000 (WB) | ProteinTech (Rosemont, MO, USA) | 10835-1-AP | 50 |
| | Phospho-ERK1/2 (T202/Y204) | rabbit polyclonal | 1:1500 (WB) | ProteinTech (Rosemont, MO, USA) | 28733-1-AP | 38/43 |
| | ERK1/2 | rabbit polyclonal | 1:3000 (WB) | Abclonal (Tokyo, Japan) | A16686 | 42/44 |
| | Phospho-JNK (Y185) | rabbit monoclonal | 1:1500 (WB) | ProteinTech (Rosemont, MO, USA) | 80024-1-RR | 43/50 |
| | JNK | rabbit polyclonal | 1:2000 (WB) | Abclonal (Tokyo, Japan) | A18678 | 43/50 |
| | c-JUN | rabbit polyclonal | 1:2000 (WB) | ProteinTech (Rosemont, MO, USA) | 24909-1-AP | 40 |
| | ACTB | mouse monoclonal | 1:15000 (WB) | Sigma-Aldrich (St Louis, MO, USA) | A1978 | 43 |
| | ACTB | rabbit polyclonal | 1:1000 (WB) | MBL (Nagoya, Japan) | PM053 | 43 |
| Secondary antibody | HRP-conjugated anti-mouse IgG | goat polyclonal | 1:15000 (WB) | ThermoFisher Scientific (Waltham, MA, USA) | 31430 | |
| | HRP-conjugated anti-rabbit IgG | goat polyclonal | 1:7500 (WB) | ThermoFisher Scientific (Waltham, MA, USA) | AP307P | |
| | Alexa Fluor 488-labeled anti-rabbit IgG | goat polyclonal | 1:1000 (ICC) | Abcam (Cambridge, UK) | ab150077 | |

WB: western blotting, ICC: immunocytochemistry, MW: molecular weight, HRP: horseradish peroxidase; MBL: Medical & Biological Laboratories
